# Obstruction of Bowels

**Published:** 1888-09

**Authors:** Thomas A. Pope

**Affiliations:** Cameron, Texas


### OBSTRUCTION OF BOWELS.

By Thomas A. Pope, M. D., Cameron, Texas.

For Daniel's Texas Medical Journal.

I WISH to call attention to the following case, as I think some of its features will interest many of the medical profession. I was called to see Mrs. X. and got the following history:

For several months her bowels had been irregular. Was generally compelled to take medicine to move the bowels, and the stools were liquid or semi-liquid. A few days before I saw her, she had a slight fever, accompanied with some nausea and vomiting. An eclectic was called in, who, after vomiting her, gave podophyllin freely, which caused large liquid stools. She had been sick about four days when I saw her. Temperature, 100 F.; conjunctiva slightly yellow; patient badly nourished; nausea persistent. On examining the abdomen, a large boggy tumor, extending from the cœcum to the lower border of the liver, could be felt and seen. There was no soreness, and the tumor was slightly movable. There was some pain and tenderness over the liver; and in this connection I will speak of the jaundice, which soon became general. This, I think, was due to the obstruction of the bile duct by the accumulation in the colon, for as the colon became emptied the jaundice disappeared without any special treatment. Naturally concluding that the tumor was caused by impacted fæces in the ascending colon, the treatment was solely directed to removing the mass. Cathartics were evidently useless, as this class of remedies had been effectually tried. There was through the mass an opening through which the contents of the upper bowel escaped.

I introduced into the bowel a tube, twelve inches long, and through this injected about half a gallon of warm water and glycerine, and at the same time applied a large poultice over the tumor. The water remained but a short time and on its return brought away a considerable mass of hard fecal matter.

I then injected into the bowel four grains of cocaine dissolved in two ounces of water, and fifteen minutes afterward placed the patient in the knee and chest position, and injected one quart of olive oil into the bowel. This oil was retained several hours, and when the bowels moved at least half a gallon of fecal matter came mixed with the oil. Most of the fecal matter was semi-liquid, of pea green color, but mixed with it was quite a large number of hard, scybalous masses, of varying sizes, of dark color, and had evidently occupied the bowel for a long period.

The enema of oil was repeated once in twenty-four hours, and a small dose of sulphate magnesia was given, per os, ten hours after the enema of oil.

Several times a day the tumor was gently but thoroughly kneaded. Under this treatment the mass slowly broke down and was carried out, and about the eighth day the tumor had entirely disappeared and the patient was convalescent.

There is perhaps nothing in this case worth reporting, but as such cases are somewhat rare, and the treatment in this case was so simple and yet effectual, I send it. There was nothing original in the treatment. I think I have somewhere read of substantially the same line of treatment being adopted, but I do not now remember where.

